# SENSE OF CONTROL AND DIGITAL LITERACY AMONG OLDER ADULTS: THE ROLE OF PERSONAL MASTERY AND PERCEIVED CONSTRAINTS

**DOI:** 10.1093/geroni/igad104.1995

**Published:** 2023-12-21

## Abstract

Digital literacy (DL) is necessary for older adults to fully participate in society in the era of digital technology. This study investigated how the sense of control (SOC) and its two internal dimensions (personal mastery (PM) and perceived constraints (PC)) interact with DL among older Chinese adults. A total of 163 older netizens aged 60 to 76 (Mean age 66.0, SD = 3.57, 72.4% female) were recruited from Beijing in 2021 through convenience sampling and followed for 1 year. DL was measured by a self-designed Digital Literacy Scale among Older Adults (Range 1-5; Cronbach’s α = 0.96) and SOC was measured by the Sense of Control Scale (Range 1-7; Cronbach’s α = 0.88). Cross-lagged analysis and half-longitudinal mediation analysis were used after adjusting for age, gender, and education. The DL were 3.51 and 3.52 at baseline and year-1 on average. Higher baseline SOC (Adjusted beta = 0.23, 95% Confidence Interval = [0.08, 0.38]) and PM (0.26 [0.10, 0.42]) were associated with higher year-1 DL. Higher baseline DL was related to reduced year-1 PC (-0.16 [-0.30, -0.02]). In addition, half-longitudinal mediation analysis showed that higher PM at baseline predicted an increase in DL from baseline to year-1 (0.29 [0.13, 0.43]), whereas higher baseline DL was related to a decrease in PC from baseline to year-1 (-0.16 [-0.30, -0.03]). Our findings indicate that the DL of older netizens is closely related to their internal factors. Older netizens might benefit from better SOC, especially personal mastery, thus narrowing the secondary digital divide.

